# Protective Effects and Potential Mechanisms of *Bacillus subtilis* Ya3.1 Against *Aeromonas hydrophila* Infection in Hybrid Sturgeon (*Acipenser schrenckii* ♂ × *Huso dauricus* ♀)

**DOI:** 10.3390/ani16121879

**Published:** 2026-06-17

**Authors:** Wei Huang, Yang Liu, Lanyin Liu, Xin Lv, Yongkang Song, Tuyan Luo

**Affiliations:** 1 Institute of Agricultural Quality Standards and Testing Technology Research, Fujian Academy of Agricultural Sciences, No. 247, Wusi Road, Gulou District, Fuzhou 350003, China; lifehuangwei@aliyun.com (W.H.); liuyang@faas.cn (Y.L.); lly87119@126.com (L.L.); lux_ing@126.com (X.L.); lifesongyongkang@126.com (Y.S.); 2 Fujian Key Laboratory of Agro-Products Quality and Safety, Fujian Academy of Agricultural Sciences, No. 247, Wusi Road, Gulou District, Fuzhou 350003, China

**Keywords:** host-derived probiotics, fish disease resistance, gut microbiota, innate humoral immunity, hepatic metabolomics

## Abstract

Bacterial disease caused by *Aeromonas hydrophila* is a major constraint in intensive sturgeon aquaculture. This study examined the intestinal, humoral, and hepatic responses associated with dietary or waterborne pretreatment with the host-derived probiotic *Bacillus subtilis* Ya3.1 during *A. hydrophila* infection in hybrid sturgeon (*Acipenser schrenckii* ♂ × *Huso dauricus* ♀). Absolute taxon-targeted qPCR, serum innate immune assays, intestinal 16S rRNA gene sequencing, and untargeted liver metabolomics were integrated to evaluate probiotic-associated protective responses. Ya3.1 pretreatment increased intestinal *B. subtilis*-associated gene copy numbers before challenge and reduced the post-challenge *A. hydrophila*-associated burden. Among surviving fish at 10 days post-infection, both probiotic-pretreated groups showed significantly higher alkaline phosphatase activity than the infected control group; the dietary pretreatment group also exhibited a significantly higher complement C3 concentration. Microbiota analysis revealed marked community restructuring in untreated challenged fish, characterized by enrichment of *Aeromonas* and other opportunistic taxa, whereas probiotic-pretreated fish showed reduced challenge-associated compositional displacement and relative enrichment of *Bacillus*-related taxa. Liver metabolomics further indicated that Ya3.1 pretreatment was associated with altered hepatic metabolic profiles, with glycerophospholipid metabolism emerging as a recurrent candidate pathway. Overall, Ya3.1-associated protection was associated with a lower pathogen burden, reduced microbiota displacement, partial preservation of humoral immune indices, and altered hepatic metabolic responses.

## Simple Summary

*Aeromonas hydrophila* infection causes serious losses in sturgeon aquaculture, and antibiotic-dependent control is increasingly undesirable. This study evaluated whether pretreatment with the host-derived probiotic *Bacillus subtilis* Ya3.1 helps hybrid sturgeon resist *A. hydrophila* infection and explored the associated intestinal, immune, and metabolic responses. Fish pretreated with Ya3.1 via feed or the rearing water showed lower intestinal *A. hydrophila*-associated loads and higher post-challenge survival than untreated infected fish. Probiotic pretreatment also reduced infection-associated gut microbiota displacement, helped maintain selected serum immune indices, particularly alkaline phosphatase activity and complement C3, and was associated with a distinct hepatic metabolic response. Glycerophospholipid metabolism was identified as a recurrent candidate pathway in both probiotic delivery routes. These results suggest that Ya3.1 may enhance disease resilience through changes associated with pathogen burden, challenge-associated microbiota restructuring, selected innate humoral indicators, and hepatic metabolism. Further studies using strain-specific tracking and functional validation are needed to confirm the underlying causal mechanisms.

## 1. Introduction

Sturgeon aquaculture is an important branch of freshwater farming because sturgeons combine broad environmental adaptability with high market value for both meat and caviar [1]. However, intensification increases the risk of opportunistic bacterial outbreaks and has become a major obstacle to sustainable production. Among these pathogens, *Aeromonas hydrophila* is particularly important because it can cause enteritis, ascites, and hemorrhagic septicemia, resulting in severe mortality and economic losses [2,3]. Disease control has traditionally relied on chemotherapeutics and antibiotics, but their extensive use in aquaculture has contributed to antimicrobial resistance and disruption of the intestinal microbiota, with negative consequences for mucosal immunity [4,5]. As prophylactic antibiotic use becomes increasingly restricted [6,7], sustainable and immunologically safe alternatives are needed. In China, hybrid sturgeon (*Acipenser schrenckii* ♂ × *Huso dauricus* ♀) has become an economically important cultured hybrid because of its favorable growth performance, stable artificial propagation, and broad use in meat and caviar production [8]. Its clearly defined parentage, consistent hatchery availability, and relevance to *A. hydrophila*-associated disease make it a suitable experimental host for evaluating antibiotic-sparing disease-control strategies in farmed sturgeons [2,3].

Probiotics are widely regarded as promising alternatives to antibiotics in aquaculture because they can improve host performance, modulate immune function, and reduce disease susceptibility [4,9,10]. Among them, *Bacillus subtilis* is attractive because its spore-forming capacity supports environmental resilience and facilitates industrial application [11,12,13]. Previous studies have shown that *B. subtilis* can limit pathogen colonization through competitive exclusion and antimicrobial metabolite production, while also modulating systemic immunity in fish [14,15,16]. Despite its widespread application, many *B. subtilis* strains used in aquaculture are derived from non-host or terrestrial sources [17]. Such strains may display variable adaptation to aquatic environments and fish intestinal niches, leading to inconsistent probiotic performance [18]. In contrast, host-associated probiotic strains isolated from the intestine of the target species may have greater niche compatibility and species-specific functional potential [18,19]. Although probiotics, prebiotics, and synbiotics have been investigated in several sturgeon species, existing studies have largely evaluated commercial or non-host-derived preparations. For instance, commercial probiotic mixtures containing *B. subtilis* have been evaluated in Siberian sturgeon (*Acipenser baerii*) and Persian sturgeon (*Acipenser persicus*) [20,21]. These studies demonstrate the potential value of probiotic supplementation in sturgeon culture, but they provide limited evidence for the efficacy of host-derived *Bacillus* strains isolated from the sturgeon intestine.

In our previous 6-week feeding trial, the autochthonous strain *B. subtilis* Ya3.1, isolated from the intestine of healthy hybrid sturgeon, improved growth performance, intestinal microbial composition, serum complement levels, and post-challenge survival following *A. hydrophila* infection [22]. These findings identified Ya3.1 as a promising probiotic candidate; however, the biological processes associated with protection during active infection remain incompletely defined. In fish, resistance to bacterial challenge is unlikely to depend on a single pathway; rather, it is associated with coordinated changes in the intestinal microbiota, innate immune defenses, and host metabolism [23,24,25]. Recent fish studies further support this systems-level view. Qiu et al. [26] showed that *Lysinibacillus sphaericus* HY3 supplementation in adult zebrafish reshaped the gut microbiota, modulated immune-related metabolites, enhanced hepatic antioxidant and immune capacity, and improved resistance to *A. hydrophila* infection. Gu et al. [27] reported that *Vibrio harveyi* infection in *Sebastes schlegelii* induced coordinated changes in gut microbial composition, metabolite profiles, inflammatory cytokine expression, and intestinal barrier-related genes. These findings support the use of integrated microbiota–immune–metabolomic analyses to investigate antibacterial immunity in fish. Most probiotic studies in sturgeon have focused on isolated or limited endpoints, such as growth performance, survival, feed utilization, blood parameters, or individual immune markers. By contrast, integrative mechanistic investigations that link probiotic-associated bacterial dynamics with pathogen burden, intestinal microbiota restructuring, immune status, and hepatic metabolic adaptation remain scarce.

Accordingly, the present study was designed as an integrative follow-up to our previous feeding trial to characterize host and microbial responses associated with Ya3.1 pretreatment during *A. hydrophila* challenge in hybrid sturgeon (*A. schrenckii* ♂ × *H. dauricus* ♀). By combining taxon-targeted bacterial qPCR, serum innate immune assays, intestinal microbiota profiling, and liver metabolomics, we aimed to identify candidate host–microbe processes associated with the Ya3.1-related protective phenotype and to generate mechanistic hypotheses for future validation.

## 2. Materials and Methods

### 2.1. Ethics Statement and Experimental Fish

All procedures complied with Chinese regulations governing the use and care of laboratory animals and were approved by the Ethics Committee of the Department of Aquatic Product Quality and Safety, Institute of Agricultural Quality Standards and Testing Technology Research, Fujian Academy of Agricultural Sciences (approval no. SYXK 2020-0007; approval date: 12 March 2024).

Hybrid sturgeon fingerlings (*A*. *schrenckii* ♂ × *H*. *dauricus* ♀) were obtained with written informed consent from a commercial aquaculture facility in Linqu County, Shandong Province, China, and were acclimated for two weeks in a recirculating aquaculture system at the Fisheries Research Laboratory, Fujian Academy of Agricultural Sciences. Upon arrival, the fish were approximately 38 days post-hatching and had recently transitioned to artificial feed; after acclimation, only clinically healthy individuals, approximately 52 days post-hatching with an initial body weight of 5.02 ± 0.22 g, were selected for the 6-week probiotic pretreatment trial based on normal swimming and feeding behavior and the absence of visible external abnormalities. The present infection challenge constituted the post-intervention challenge-response phase of the same fish cohort used in our previous 6-week probiotic trial [22]. Previously reported data are used here only for contextual comparison and are explicitly distinguished from the newly generated analyses presented in this manuscript. Briefly, fish were maintained in an indoor recirculating aquaculture system and randomly allocated to three experimental groups (three replicate tanks per group): (1) control group (CK), fed a commercial basal diet; (2) dietary probiotic group (BF), fed the basal diet supplemented with 1.0 × 10^8^ CFU g^−1^ *B. subtilis* Ya3.1; and (3) waterborne probiotic group (BW), fed the basal diet with *B. subtilis* Ya3.1 added to the rearing water every 48 h to maintain 1.0 × 10^7^ CFU L^−1^. Throughout the six-week pretreatment period, fish were hand-fed the experimental diets to apparent satiation twice daily at 09:00 and 17:00, and water quality remained stable, with temperature at 16–20 °C, dissolved oxygen > 6 mg L^−1^, pH 6.8–7.4, and total ammonia nitrogen and nitrite < 0.1 mg L^−1^. At the end of pretreatment, all fish were clinically normal and were subsequently subjected to bacterial challenge.

### 2.2. Bacterial Culture

#### 2.2.1. Probiotic Strain

The host-derived probiotic strain *B. subtilis* Ya3.1 was originally isolated from the intestinal tract of healthy hybrid sturgeon and deposited at the China General Microbiological Culture Collection Center (CGMCC 35019). The culture conditions, fermentation procedure, spray-drying process, and preparation of the probiotic powder (1.4 × 10^12^ CFU g^−1^ viable cells) were described previously [22].

#### 2.2.2. Pathogenic Strain

The pathogenic strain *A. hydrophila* B3, previously isolated and confirmed to be virulent to hybrid sturgeon by the Fisheries Research Laboratory of the Fujian Academy of Agricultural Sciences, was used for the challenge experiment. The strain was revived from −80 °C glycerol stocks and cultured in tryptic soy broth (TSB) at 28 °C for 24 h with shaking at 150 rpm. Cells were harvested by centrifugation at 4000× *g* for 10 min at 4 °C, washed twice with sterile phosphate-buffered saline (PBS, pH 7.4), and resuspended in PBS. The final concentration was adjusted to 1.0 × 10^8^ CFU mL^−1^ using a pre-established OD600–CFU calibration curve and verified by serial dilution followed by plate counting on tryptic soy agar (TSA).

### 2.3. Challenge Test and Experimental Design

At the end of the 6-week pretreatment period, nine fish from each group (three per replicate tank) were randomly sampled before challenge to establish baseline physiological profiles (CK, BF, and BW).

For the infection challenge, an additional 60 visually healthy fish were selected from each pretreatment group, with equal representation from each replicate tank (20 fish per tank; three tanks per treatment). The mean body weight was 21.3 ± 2.8 g. Fish were anesthetized with MS-222 (60 mg L^−1^; Merck, Darmstadt, Germany) and intraperitoneally injected with 200 μL of *A. hydrophila* B3 suspension (1.0 × 10^8^ CFU mL^−1^), corresponding to 2.0 × 10^7^ CFU fish^−1^. This dose corresponded to the LD_50_ previously established for this host–pathogen system in a preliminary study [22]. Post-challenge fish derived from CK, BF, and BW were designated as ACK, ABF, and ABW, respectively.

To evaluate whether protection persisted after pretreatment rather than continued dosing, a withdrawal design was used after the challenge. The rationale for this design was to determine whether the protective effects induced by the 6-week Ya3.1 pretreatment could persist after discontinuation, rather than depending on continuous probiotic administration during active infection. This approach also minimized the immediate influence of newly supplied probiotic cells on pathogen growth, host responses, and the rearing environment after challenge. After the challenge, all fish were maintained in their original replicate tanks, with husbandry management and environmental conditions kept consistent with those used during the pretreatment period. However, all groups received the unsupplemented basal diet only, and probiotic administration was discontinued. Mortality and clinical signs were recorded daily for 10 days post-infection (dpi), and dead fish were promptly removed. Survival was monitored for the full 10-day period, after which terminal sampling was performed at 10 dpi. Fish euthanized at 10 dpi for tissue collection were counted as terminal survivors in the cumulative survival analysis.

### 2.4. Sample Collection and Processing

Fish were fasted for 24 h before each sampling to standardize digestive status among groups and to reduce the acute effects of recently ingested feed, residual digesta, and feed-associated transient bacteria on luminal microbiota profiles. At baseline (CK, BF, and BW) and at terminal post-challenge sampling (10 dpi; ACK, ABF, and ABW), three visually healthy fish were randomly collected from each replicate tank and deeply anesthetized with MS-222 (100 mg L^−1^). Because only surviving fish were available at 10 dpi, all post-challenge datasets should be interpreted as survivor-associated terminal profiles and may not represent responses in fish that succumbed to infection before terminal sampling.

Blood was collected from the caudal vein using sterile syringes without anticoagulant, allowed to clot at 4 °C for 4–6 h, and centrifuged at 3000× *g* for 10 min at 4 °C. Serum was harvested, aliquoted, flash-frozen in liquid nitrogen, and stored at −80 °C for analysis of innate immune indices. Samples from three fish collected per tank were processed individually.

After blood collection, two of the three fish sampled per tank were randomly designated for tissue collection (*n* = 6 per group). Fish were aseptically dissected on a sterile bench. Liver tissue was rapidly excised, flash-frozen, and stored at −80 °C for untargeted metabolomics. For intestinal analyses, spiral valve luminal contents were collected aseptically using sterile instruments that were changed between fish to prevent cross-contamination. Aliquots of the same intestinal samples were used for both targeted bacterial qPCR and 16S rRNA gene sequencing.

### 2.5. Absolute Quantification of Targeted Bacteria

To estimate the probiotic- and pathogen-associated bacterial loads in the intestine, absolute quantitative real-time PCR (qPCR) was performed using manufacturer-validated taxon-targeted assays for *B. subtilis* and *A. hydrophila*. Total genomic DNA was extracted from precisely weighed spiral valve luminal contents using the QIAamp Fast DNA Stool Mini Kit (Qiagen, Düsseldorf, Germany), according to the manufacturer’s instructions. DNA integrity was verified by agarose gel electrophoresis, and the concentration was measured using a Qubit 3.0 Fluorometer with the dsDNA HS Assay Kit (Invitrogen, Carlsbad, CA, USA).

Taxon-targeted qPCR assays were performed using commercial fluorescence-based quantitative PCR kits supplied by Beijing Tianjingsha Gene Technology Co., Ltd. (Beijing, China; catalog No. 14-25450 for *B. subtilis* and catalog No. 14-25310 for *A. hydrophila*). The assays targeted a *B. subtilis*-specific 16S rRNA gene region and the aerA gene of *A. hydrophila*, respectively. Reactions were performed using a Bio-Rad CFX96 Touch Real-Time PCR Detection System (Bio-Rad, Hercules, CA, USA). Standard curves were generated using 10-fold serial dilutions (10^1^–10^6^ copies μL^−1^) of the kit-provided positive controls, and the cycle threshold (Ct) values were converted into absolute copy numbers accordingly. Amplification efficiencies ranged from 95% to 105%, and all standard curves showed *R*^2^ > 0.99. The results were expressed as gene copies per ng of total extracted DNA. Because the *B. subtilis* assay was taxon-targeted rather than Ya3.1-specific, these data are interpreted as *B. subtilis*-associated copy numbers and cannot distinguish the persistence of the administered strain from the expansion of closely related indigenous *Bacillus* populations.

### 2.6. Serum Innate Immune Parameters

Serum activities of alkaline phosphatase (AKP), acid phosphatase (ACP), lysozyme (LZM), and serum concentrations of complement component 3 (C3) and complement component 4 (C4) were measured using commercial assay kits (Nanjing Jiancheng Bioengineering Institute, Nanjing, China). Catalog numbers were AKP (A059-2-2), ACP (A060-2-2), LZM (A050-1-1), C3 (H186-1-2), and C4 (H186-2-1). All assays were performed in triplicate according to the manufacturer’s instructions, and absorbance was measured using a SpectraMax i3x microplate reader (Molecular Devices, San Jose, CA, USA).

### 2.7. Intestinal Microbiota Analysis

Total genomic DNA extracted from the intestinal samples described in Section 2.5 was used for 16S rRNA gene sequencing. The V3–V4 region of the bacterial 16S rRNA gene was amplified with primers 341F (5′-CCTACGGGNGGCWGCAG-3′) and 805R (5′-GACTACHVGGGTATCTAATCC-3′). PCR amplification, library preparation, and sequencing were performed by Sangon Biotech Co., Ltd. (Shanghai, China) using an Illumina NextSeq 2000 platform (Illumina, San Diego, CA, USA). Raw reads were deposited in the NCBI Sequence Read Archive under BioProjects PRJNA1370269 and PRJNA1455859, respectively.

Sequencing reads were demultiplexed and quality filtered. Primer and adapter sequences were removed using Cutadapt (v1.18). Paired-end reads were merged with PEAR (v0.9.8), and reads with mean Phred quality score < 20 were discarded using fqtrim (v0.94). Chimeras were removed with USEARCH (v11.0.667). Across the intestinal samples included in the 16S rRNA gene sequencing dataset, 45,591–307,758 raw reads were obtained per sample. Following the complete preprocessing workflow, 39,392–267,880 clean reads per sample were retained, corresponding to read-retention rates of 64.98–99.80%. Good’s coverage values ranged from 99.81% to 99.99%, indicating that the sequencing depth was sufficient to capture the majority of bacterial diversity in these intestinal samples. Sample-specific sequencing quality metrics are provided in Supplementary Table S1. For consistency with the analytical workflow used across all intestinal samples in this study, high-quality reads were clustered into operational taxonomic units (OTUs) at 97% sequence similarity, and representative sequences were assigned taxonomy using the RDP classifier (v2.12) against the SILVA database (v138), with a confidence threshold of 0.8.

For alpha diversity analysis, the OTU table was rarefied to the minimum sequencing depth across samples. Observed OTUs (Sobs), Chao1, ACE, Shannon, Simpson, and Good’s coverage were calculated using Mothur (v1.43.0), and rarefaction curves were generated at 3% genetic distance. Community compositional variation was visualized using principal component analysis (PCA) based on normalized OTU-level relative abundances, and beta diversity was assessed using principal coordinates analysis (PCoA) based on Bray–Curtis dissimilarities calculated in QIIME 2 (v2024). Group differences in overall community structure were assessed using PERMANOVA with 999 permutations implemented in the adonis2 function of the R package vegan v2.5-6, followed by pairwise comparisons with Benjamini–Hochberg false discovery rate (FDR) correction; FDR-adjusted *p* values < 0.05 were considered significant. To quantify challenge-associated microbiota displacement, Bray–Curtis distances were calculated from each post-challenge sample to the centroid of its corresponding pre-challenge baseline group, namely ACK to the CK centroid, ABF to the BF centroid, and ABW to the BW centroid. Differences in multivariate dispersion were further evaluated using PERMDISP via the betadisper approach based on the Bray–Curtis distance matrix. Differentially abundant taxa were identified using LEfSe in temporal comparisons (CK vs. ACK, BF vs. ABF, and BW vs. ABW) and between-treatment comparisons among challenged groups (ACK vs. ABF and ACK vs. ABW). High-confidence discriminatory taxa were defined using a conservative threshold of LDA > 4.0 and FDR-adjusted *p* < 0.05. This stringent criterion was used to minimize overinterpretation of weakly discriminant or low-abundance taxa in a relatively small but balanced microbiota dataset; taxa below this threshold were therefore not considered absent or biologically irrelevant, but were not emphasized as LEfSe biomarkers.

### 2.8. Hepatic Metabolomics Analysis

Liver tissue samples (50 ± 5 mg) were homogenized in 400 μL of cold extraction solvent (methanol/water, 4:1, *v*/*v*) containing 0.02 mg mL^−1^ L-2-chlorophenylalanine as an internal standard using a Wonbio-96c tissue grinder (Shanghai Wanbo Biotechnology Co., Ltd., Shanghai, China) at 50 Hz for 6 min at −10 °C. Homogenates were sonicated at 40 kHz for 30 min at 5 °C, incubated at −20 °C for 30 min to precipitate proteins, and centrifuged at 13,000× *g* for 15 min at 4 °C. The supernatants were transferred to autosampler vials for LC–MS/MS analysis.

Metabolic profiling was performed by Majorbio Bio-Pharm Technology Co., Ltd. (Shanghai, China) using an SCIEX UPLC-Triple TOF 5600 system (AB SCIEX, Framingham, MA, USA) equipped with an ACQUITY HSS T3 column (100 mm × 2.1 mm, 1.8 μm; Waters, Milford, MA, USA). The mobile phase consisted of solvent A (water/acetonitrile, 95:5, *v*/*v*, with 0.1% formic acid) and solvent B (acetonitrile/isopropanol/water, 47.5:47.5:5, *v*/*v*/*v*, with 0.1% formic acid). Gradient elution was performed at 0.40 mL min^−1^ with a 10 μL injection volume and a column temperature of 40 °C. Mass spectrometry was conducted in both positive and negative electrospray ionization modes. The key parameters were as follows: scan range, 50–1200 *m*/*z*; ion source gases 1 and 2, 50 psi; curtain gas, 35 psi; source temperature, 500 °C; ion spray voltage floating, 5500 V in positive mode and −4500 V in negative mode; declustering potential, 80 V; and collision energy, 40 ± 20 eV. Quality control samples prepared by pooling equal aliquots of all extracts were injected every six experimental samples. The relative standard deviations of the internal standards and representative metabolites in the QC samples were <30%, indicating acceptable analytical stability.

Raw LC–MS data were processed using Progenesis QI v3.0 (Waters Corporation, Milford, MA, USA) for data preprocessing, including baseline filtering, peak detection, peak integration, retention-time correction, and peak alignment. A data matrix containing retention time, mass-to-charge ratio (*m*/*z*), and peak intensity was generated for subsequent analysis. Peaks corresponding to internal standards, background noise, and column bleed were removed, and redundant peaks were merged. Metabolite features were annotated in Progenesis QI by matching MS and MS/MS spectral information against the Human Metabolome Database (HMDB, version 5.0; http://www.hmdb.ca/ (accessed on 13 August 2025)), METLIN (2019 version; https://metlin.scripps.edu/ (accessed on 13 August 2025)), and an in-house MJDB database (Majorbio). The database-matching criteria were set as follows: precursor mass tolerance < 10 ppm, fragment mass tolerance < 0.02 Da, and MS/MS match score > 30 based on the Progenesis QI score. PLS-DA and OPLS-DA analyses were performed using the ropls package (v1.6.2) in R and were used as exploratory supervised models rather than as stand-alone evidence of discrimination. Fold changes were calculated for all pairwise comparisons and displayed in the volcano plots to indicate the direction and magnitude of metabolite changes; however, no fixed fold-change cutoff was applied as an additional exclusion criterion. Significant differential metabolites (SDMs) were defined as metabolites with VIP > 1.0 in the OPLS-DA model and FDR-adjusted *p* < 0.05 in the Student’s *t*-test. SDMs were mapped to KEGG pathways, and pathway enrichment was tested using Fisher’s exact test implemented in scipy.stats (v1.7.3). Pathways with FDR-adjusted *p* < 0.05 were considered significantly enriched. All metabolomics data visualizations were generated using the Majorbio Cloud Platform.

### 2.9. Statistical Analysis

Statistical analyses were performed using SPSSPRO (https://www.spsspro.com/ (accessed on 12 January 2026)) unless otherwise stated. Variables used for group mean comparisons are presented as the mean ± standard error (SE). Potential tank-origin effects were addressed according to the structure of each dataset. For serum immune parameters and targeted qPCR data, the tank was treated as the experimental unit; measurements from fish sampled within the same tank were averaged before inferential analysis, resulting in three replicate tanks per treatment (*n* = 3). For microbiota sequencing and untargeted metabolomics, two fish per tank were included in each treatment to maintain equal tank representation and reduce potential confounding by tank origin. Individual fish profiles were analyzed as biological observations, and treatment effects were interpreted under this balanced tank-origin design.

Normality and homogeneity of variance were assessed using the Shapiro–Wilk and Levene tests, respectively, where applicable. For low-dimensional comparisons, including serum immune parameters, targeted qPCR data, alpha-diversity indices, and taxon-level relative abundances, normally distributed data with homogeneous variances were analyzed using one-way ANOVA followed by Tukey’s HSD post hoc test. When these assumptions were not met, the Kruskal–Wallis test followed by Dunn’s post hoc test with Benjamini–Hochberg false discovery rate (FDR) correction was applied. For post hoc pairwise comparisons, statistical significance was defined as a multiplicity-adjusted *p* value < 0.05. Cumulative survival was estimated using the Kaplan–Meier method, and overall and pairwise differences were evaluated using log-rank tests with Bonferroni adjustment; Bonferroni-adjusted *p* values < 0.05 were considered significant. For microbiome and metabolome analyses involving multiple testing, *p* values were adjusted using the Benjamini–Hochberg FDR procedure. This correction was applied to pairwise PERMANOVA comparisons, LEfSe taxonomic biomarker analyses, metabolite-level tests, and KEGG pathway-enrichment analyses, with significance defined as an FDR-adjusted *p* value < 0.05. Bray–Curtis centroid-displacement values among challenged groups were compared using the Kruskal–Wallis test followed by pairwise Mann–Whitney U tests with Holm correction, and Holm-adjusted *p* values < 0.05 were considered significant. PERMDISP significance was assessed by permutation testing, with permutation *p* values < 0.05 considered significant.

## 3. Results

### 3.1. Effect of B. subtilis Ya3.1 on Resistance Against A. hydrophila Infection

**Figure 1:**
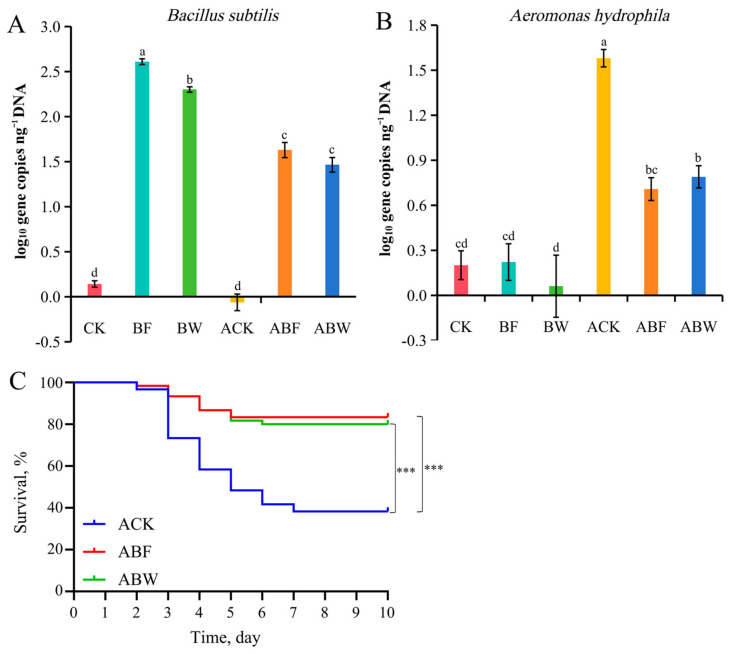
Protective effects of *B. subtilis* Ya3.1 pretreatment against *A. hydrophila* challenge in hybrid sturgeon. (**A**) Intestinal *B. subtilis*-associated gene copy numbers. (**B**) Intestinal *A. hydrophila*-associated gene copy numbers. (**C**) Kaplan–Meier cumulative survival curves of hybrid sturgeon following *A. hydrophila* challenge over a 10-day observation period. Data in panels (**A**,**B**) are presented as the mean ± SE of log10-transformed gene copy numbers (*n* = 3 tanks per group). Different lowercase letters indicate significant differences among groups (adjusted *p* < 0.05). Asterisks in panel (**C**) indicate significant differences compared with the ACK group (*** adjusted *p* < 0.001). Abbreviations: CK, unchallenged control group; BF, unchallenged dietary *B. subtilis* Ya3.1-pretreated group; BW, unchallenged waterborne *B. subtilis* Ya3.1-pretreated group; ACK, *A. hydrophila*-challenged control group; ABF, *A. hydrophila*-challenged dietary *B. subtilis* Ya3.1-pretreated group; ABW, *A. hydrophila*-challenged waterborne *B. subtilis* Ya3.1-pretreated group.

The absolute abundances of the targeted bacterial markers and cumulative survival after *A. hydrophila* challenge are shown in Figure 1. Before infection, both dietary (BF) and waterborne (BW) pretreatment with *B. subtilis* Ya3.1 were associated with significantly higher intestinal *B. subtilis*-associated gene copy numbers than the CK group (Figure 1A). After challenge, the intestinal *A. hydrophila*-associated burden was significantly lower in the ABF and ABW groups than in the ACK group (Figure 1B). Consistent with these differences, prior exposure to Ya3.1 significantly improved the cumulative survival rate after infection (Figure 1C). Kaplan–Meier analysis showed an overall group effect (log-rank *p* < 0.0001), and Bonferroni-adjusted pairwise comparisons indicated that both probiotic-pretreated groups had significantly higher survival probabilities (83.3% and 80.0%) than ACK (38.3%; *p* < 0.001 for both comparisons). Together, these data indicate that Ya3.1 pretreatment was associated with increased intestinal *B. subtilis*-related signals before challenge, a lower pathogen-associated burden after challenge, and improved survival.

### 3.2. Serum Innate Humoral Responses to A. hydrophila Challenge

**Figure 2:**
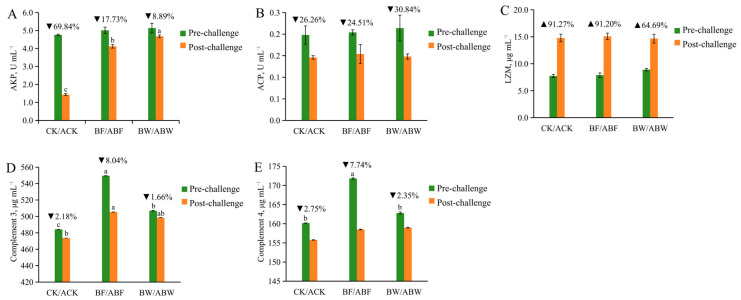
Effects of *B. subtilis* Ya3.1 pretreatment on serum innate immune parameters before and after *A. hydrophila* challenge. (**A**) AKP activity. (**B**) ACP activity. (**C**) LZM activity. (**D**) C3 concentration. (**E**) C4 concentration. Data are presented as the mean ± SE (*n* = 3 tanks per group). Different lowercase letters indicate significant differences among groups at the same sampling stage (adjusted *p* < 0.05). Triangles and percentages indicate post-challenge changes relative to the corresponding pre-challenge baseline. Abbreviations: AKP, alkaline phosphatase; ACP, acid phosphatase; LZM, lysozyme; C3, complement component 3; C4, complement component 4; CK, unchallenged control group; BF, unchallenged dietary *B. subtilis* Ya3.1-pretreated group; BW, unchallenged waterborne *B. subtilis* Ya3.1-pretreated group; ACK, *A. hydrophila*-challenged control group; ABF, *A. hydrophila*-challenged dietary *B. subtilis* Ya3.1-pretreated group; ABW, *A. hydrophila*-challenged waterborne *B. subtilis* Ya3.1-pretreated group.

The effects of Ya3.1 pretreatment on serum innate immune indices before challenge and in surviving fish at 10 dpi are shown in Figure 2A–E. At baseline, AKP, ACP, and LZM activities were comparable among CK, BF, and BW (Figure 2A–C). In contrast, complement-related indices responded to Ya3.1 pretreatment: serum C3 concentration was significantly higher in both BF and BW than in the CK group, whereas C4 concentration was significantly elevated only in BF relative to CK (Figure 2D,E). At 10 dpi, AKP, ACP, C3, and C4 generally declined relative to their corresponding pre-challenge levels (Figure 2A,B,D,E), while LZM activity tended to increase (Figure 2C). Despite these challenge-associated shifts, ABF and ABW maintained significantly higher AKP activity than ACK (Figure 2A). For C3, ABF showed a significantly higher concentration than ACK, whereas ABW displayed an intermediate level that did not differ significantly from either ACK or ABF (Figure 2D). Collectively, these results indicate that Ya3.1 pretreatment partially preserved selected humoral immune indices in surviving fish after challenge, particularly AKP activity and, more prominently under dietary pretreatment, C3 concentration.

### 3.3. Modulation of the Intestinal Microbiota During A. hydrophila Infection

#### 3.3.1. Effects on Intestinal Microbial Diversity

Alpha diversity indices of the intestinal microbiota are summarized in Table 1. Richness estimators (Sobs, Chao1, and ACE) tended to increase after challenge but did not differ significantly among the groups. In contrast, diversity-related indices (Shannon and Simpson) showed significant changes in the untreated challenged group. Specifically, ACK showed a significantly lower Simpson index than CK and BF (0.26 ± 0.02 vs. 0.62 ± 0.05) and a significantly higher Shannon index than CK (2.01 ± 0.11 vs. 0.80 ± 0.07). By comparison, ABF and ABW showed intermediate Simpson values (0.57 ± 0.08 and 0.50 ± 0.08, respectively) and Shannon values (1.14 ± 0.22 and 1.36 ± 0.23, respectively), and they were not significantly separated from their corresponding pre-challenge groups. These results indicate that *A. hydrophila* challenge was associated with altered community diversity in untreated fish, while probiotic-pretreated fish retained community diversity profiles comparable to their pre-challenge baselines.

**Figure 3:**
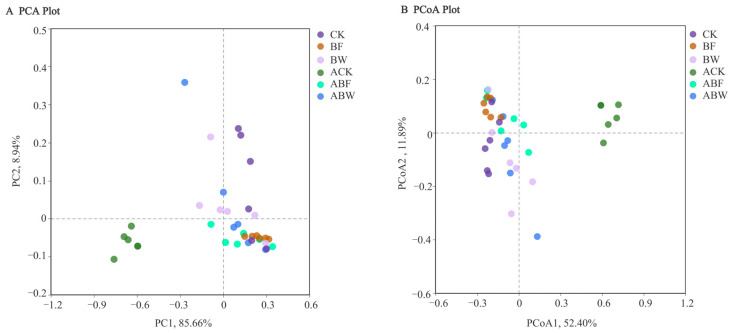
Beta-diversity ordination of intestinal microbial communities across experimental groups. (**A**) PCA based on the normalized OTU-level relative abundance matrix. (**B**) PCoA based on Bray–Curtis dissimilarities. Each point represents one intestinal sample, with two fish sampled from each replicate tank (*n* = 6 fish per group). Abbreviations: PCA, principal component analysis; PCoA, principal coordinates analysis; OTU, operational taxonomic unit; CK, unchallenged control group; BF, unchallenged dietary *B. subtilis* Ya3.1-pretreated group; BW, unchallenged waterborne *B. subtilis* Ya3.1-pretreated group; ACK, *A. hydrophila*-challenged control group; ABF, *A. hydrophila*-challenged dietary *B. subtilis* Ya3.1-pretreated group; ABW, *A. hydrophila*-challenged waterborne *B. subtilis* Ya3.1-pretreated group.

Beta-diversity analyses revealed marked differences in intestinal microbial community composition among the groups (Figure 3A,B). In the PCA plot based on normalized OTU-level relative abundances (Figure 3A), ACK samples were clearly separated from the other groups, indicating a pronounced challenge-induced shift in untreated fish. A similar separation pattern was observed in the Bray–Curtis PCoA plot (Figure 3B). Consistently, PERMANOVA revealed a significant overall difference among the six groups (R^2^ = 0.769, *p* = 0.001; Supplementary Table S2). Pairwise comparisons further demonstrated that ACK differed significantly from CK, BF, BW, ABF, and ABW (all adjusted *p* < 0.05), confirming substantial microbial restructuring following the challenge in the absence of probiotic pretreatment. In contrast, the post-challenge probiotic groups tended to cluster near their corresponding pre-challenge groups. Specifically, ABW did not differ significantly from BW (adjusted *p* = 0.845), and ABF did not differ significantly from BF (adjusted *p* = 0.090). To further quantify this pattern, we calculated Bray–Curtis distances from each post-challenge sample to its corresponding pre-challenge group centroid. ACK showed a markedly greater centroid displacement than ABF and ABW (0.8644 ± 0.0251 vs. 0.2202 ± 0.0507 and 0.2154 ± 0.0509, respectively; Kruskal–Wallis *p* = 0.00332; Supplementary Table S3). In contrast, PERMDISP detected no overall difference in within-group multivariate dispersion among the six groups (overall permutation *p* = 0.5598; Supplementary Table S3). These results indicate that Ya3.1 pretreatment reduced challenge-associated compositional displacement of the intestinal microbiota, although it did not significantly alter within-group dispersion.

#### 3.3.2. Effects on Intestinal Microbial Composition

**Figure 4:**
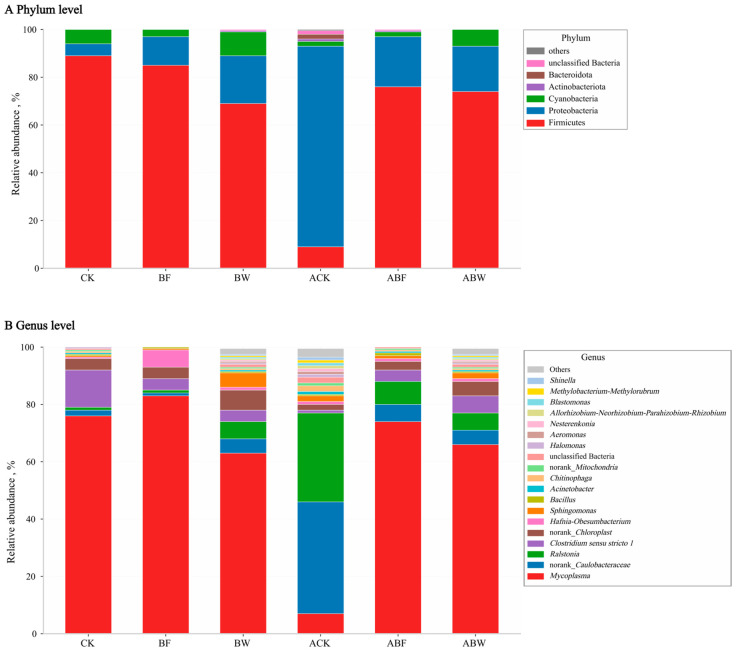
Taxonomic composition of the intestinal microbiota across experimental groups. Relative abundance of intestinal bacterial taxa at the phylum (**A**) and genus (**B**) levels. Each stacked bar represents the mean relative abundance of taxa within each group. Abbreviations: CK, unchallenged control group; BF, unchallenged dietary *B. subtilis* Ya3.1-pretreated group; BW, unchallenged waterborne *B. subtilis* Ya3.1-pretreated group; ACK, *A. hydrophila*-challenged control group; ABF, *A. hydrophila*-challenged dietary *B. subtilis* Ya3.1-pretreated group; ABW, *A. hydrophila*-challenged waterborne *B. subtilis* Ya3.1-pretreated group.

At the phylum level (Figure 4A), Firmicutes, Proteobacteria, and Cyanobacteria were the predominant taxa (relative abundance > 1%) in all pre-challenge groups, accounting for 96.31–99.91% of the sequences. Following bacterial challenge, ACK exhibited a significant decrease in the relative abundance of Firmicutes and significant increases in the relative abundances of Proteobacteria, Actinobacteriota, and Bacteroidota, indicating a shift from a Firmicutes-dominated profile to a more taxonomically heterogeneous community. No significant differences at the phylum level were detected between BF and ABF or between BW and ABW. At the genus level (Figure 4B), CK was dominated mainly by *Mycoplasma*, norank_*Caulobacteraceae*, *Ralstonia*, *Clostridium sensu stricto* 1, and norank_*Chloroplast*. Following challenge, ACK displayed an expanded set of dominant genera, including *Hafnia-Obesumbacterium*, *Acinetobacter*, *Chitinophaga*, norank_*Mitochondria*, unclassified Bacteria, *Halomonas*, *Aeromonas*, and *Nesterenkonia*. In contrast, the probiotic-pretreated groups showed less extensive challenge-associated changes in dominant genera. These compositional data indicate that Ya3.1 pretreatment was associated with reduced challenge-associated restructuring of the intestinal microbiota.

**Figure 5:**
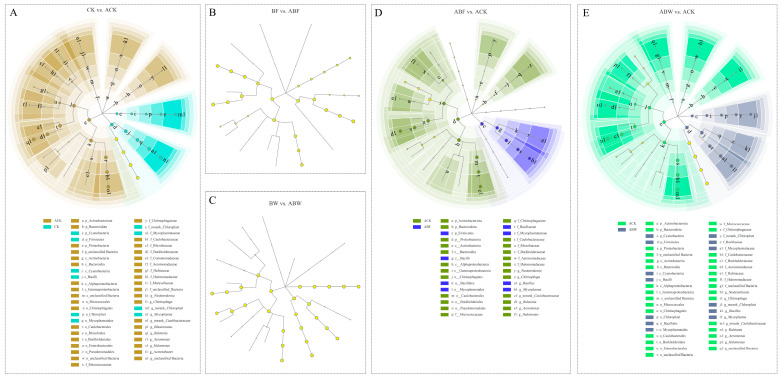
LEfSe analysis of differential intestinal bacterial taxa across pairwise group comparisons. (**A**) CK vs. ACK. (**B**) BF vs. ABF. (**C**) BW vs. ABW. (**D**) ABF vs. ACK. (**E**) ABW vs. ACK. Concentric rings in the cladogram represent taxonomic levels from kingdom to genus. Node diameter reflects relative abundance; colored nodes indicate taxa enriched in the corresponding groups, whereas yellow nodes indicate non-significant taxa. Discriminative taxa were identified using LEfSe with LDA score > 4.0 and FDR-adjusted *p* < 0.05. Abbreviations: LEfSe, linear discriminant analysis effect size; LDA, linear discriminant analysis; FDR, false discovery rate; CK, unchallenged control group; BF, unchallenged dietary *B. subtilis* Ya3.1-pretreated group; BW, unchallenged waterborne *B. subtilis* Ya3.1-pretreated group; ACK, *A. hydrophila*-challenged control group; ABF, *A. hydrophila*-challenged dietary *B. subtilis* Ya3.1-pretreated group; ABW, *A. hydrophila*-challenged waterborne *B. subtilis* Ya3.1-pretreated group.

LEfSe analysis further supported the challenge-associated restructuring of the intestinal microbiota in ACK relative to CK (Figure 5A). Compared with CK, ACK was characterized by the relative depletion of *Mycoplasma* and norank_*Chloroplast* and the enrichment of unclassified_Bacteria and several opportunistic or potentially pathogenic taxa, including *Nesterenkonia*, *Chitinophaga*, norank_*Caulobacteraceae*, *Blastomonas*, *Ralstonia*, *Aeromonas*, *Halomonas*, and *Acinetobacter*. Under the selected LEfSe threshold, no discriminatory genera were detected between BF and ABF or between BW and ABW (Figure 5B,C), consistent with greater resistance to challenge-associated compositional restructuring in the Ya3.1-pretreated groups. Direct comparisons among challenged groups showed that ABF and ABW had lower relative abundances of several ACK-enriched opportunistic taxa, whereas *Bacillus*-related lineages were relatively enriched (Figure 5D,E). Overall, these results suggest that Ya3.1 pretreatment was associated with reduced challenge-associated restructuring of the intestinal microbial community and with reduced enrichment of several opportunistic taxa in surviving fish.

### 3.4. Alterations in Hepatic Metabolic Profiles

#### 3.4.1. Multivariate Analysis of Liver Metabolomic Profiles

**Figure 6:**
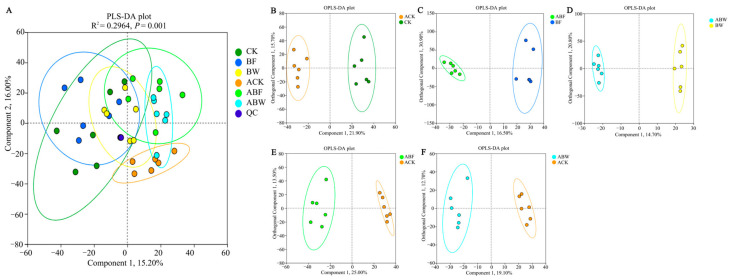
Multivariate score plots of hepatic metabolite profiles across experimental groups. (**A**) PLS-DA score plot of all groups. (**B**–**D**) OPLS-DA score plots comparing post-challenge groups with their corresponding pre-challenge groups: ACK vs. CK, ABF vs. BF, and ABW vs. BW. (**E**,**F**) OPLS-DA score plots comparing probiotic-pretreated challenged groups with the challenged control: ABF vs. ACK and ABW vs. ACK. Each point represents one liver sample, with two fish sampled from each replicate tank (*n* = 6 fish per group). Abbreviations: PLS-DA, partial least squares discriminant analysis; OPLS-DA, orthogonal partial least squares discriminant analysis; CK, unchallenged control group; BF, unchallenged dietary *B. subtilis* Ya3.1-pretreated group; BW, unchallenged waterborne *B. subtilis* Ya3.1-pretreated group; ACK, *A. hydrophila*-challenged control group; ABF, *A. hydrophila*-challenged dietary *B. subtilis* Ya3.1-pretreated group; ABW, *A. hydrophila*-challenged waterborne *B. subtilis* Ya3.1-pretreated group.

PLS-DA and OPLS-DA were applied as exploratory supervised methods to visualize overall differences in hepatic metabolite profiles among groups (Figure 6). In the global PLS-DA score plot, the six groups showed group-dependent distribution patterns, although partial overlap was observed among some groups (Figure 6A). Pairwise OPLS-DA score plots further revealed a clear separation between challenged fish and their corresponding pre-challenge groups, including ACK vs. CK, ABF vs. BF, and ABW vs. BW (Figure 6B–D). These results suggest challenge-associated shifts in hepatic metabolic profiles. In addition, the challenged probiotic-treated groups, ABF and ABW, were clearly separated from ACK in the OPLS-DA plots (Figure 6E,F), suggesting that prior Ya3.1 exposure was associated with a distinct post-challenge hepatic metabolic profile.

#### 3.4.2. Differential Metabolite Analysis

**Figure 7:**
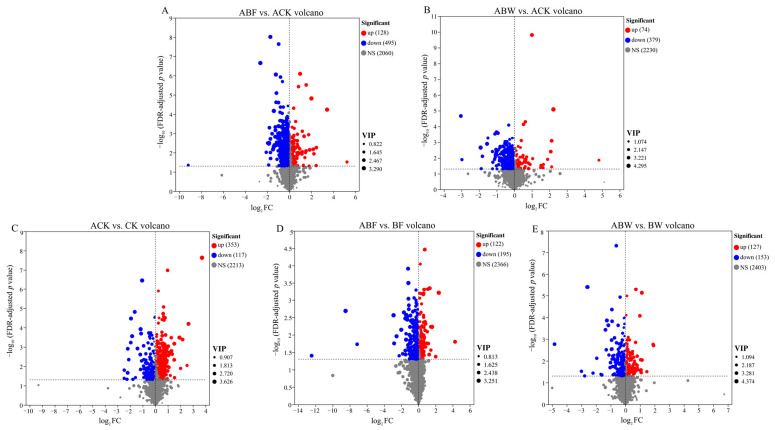
Volcano plots of hepatic SDMs across pairwise comparisons. (**A**,**B**) ABF vs. ACK and ABW vs. ACK. (**C**–**E**) ACK vs. CK, ABF vs. BF, and ABW vs. BW. Significant differential metabolites were defined as metabolites with VIP > 1.0 and FDR-adjusted *p* < 0.05. Red, blue, and grey dots denote increased, decreased, and non-significant metabolites, respectively. Abbreviations: NS, not significant; FC, fold change; VIP, variable importance in projection; FDR, false discovery rate; CK, unchallenged control group; BF, unchallenged dietary *B. subtilis* Ya3.1-pretreated group; BW, unchallenged waterborne *B. subtilis* Ya3.1-pretreated group; ACK, *A. hydrophila*-challenged control group; ABF, *A. hydrophila*-challenged dietary *B. subtilis* Ya3.1-pretreated group; ABW, *A. hydrophila*-challenged waterborne *B. subtilis* Ya3.1-pretreated group.

Differential metabolite screening was performed using VIP > 1.0 and FDR-adjusted *p* < 0.05 (Figure 7). After challenge, 623 SDMs were detected in ABF vs. ACK and 453 in ABW vs. ACK (Figure 7A,B), indicating substantial differences between probiotic-pretreated and untreated challenged fish. Within the non-supplemented group, infection was associated with 470 SDMs in ACK vs. CK (Figure 7C). The corresponding within-group comparisons yielded 317 SDMs in ABF vs. BF and 280 in ABW vs. BW (Figure 7D,E). Although SDM counts alone do not directly indicate biological severity or metabolic homeostasis, the lower number of within-group post-challenge SDMs in ABF vs. BF and ABW vs. BW is consistent with a less extensive hepatic metabolic shift in probiotic-pretreated fish.

To characterize the functional composition of the SDMs, KEGG-annotated metabolites were assigned to major compound classes, including lipids, amino acids, carbohydrates, vitamins/cofactors, and other annotated classes (Supplementary Table S4). Lipids were the predominant class across comparisons, accounting for 55.6–85.1% of the KEGG-annotated SDMs. In the direct post-challenge comparisons, ABF vs. ACK and ABW vs. ACK each contained 42 lipid-classified SDMs, representing 62.7% and 72.4% of the KEGG-annotated SDMs, respectively. Phospholipids were the largest lipid subclass in both comparisons, indicating that Ya3.1-associated hepatic metabolic differences after *A. hydrophila* challenge were mainly concentrated in lipid metabolism, particularly phospholipid metabolism.

#### 3.4.3. Effects on KEGG Metabolic Pathways

**Figure 8:**
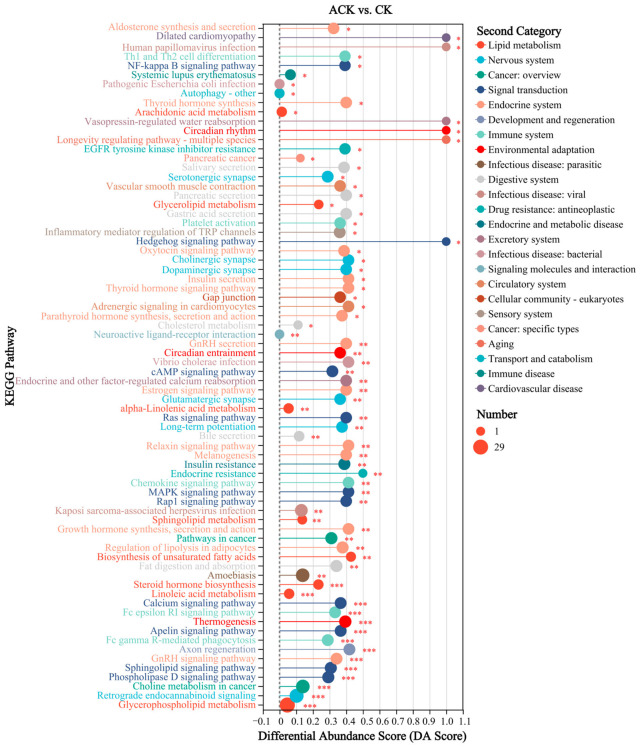
KEGG pathway enrichment analysis of hepatic SDMs in ACK versus CK after challenge. Dot size indicates the number of mapped metabolites, and colors indicate KEGG second-level categories. Positive and negative DA scores indicate overall metabolite increases and decreases in ACK relative to CK, respectively. Asterisks denote FDR-adjusted pathway enrichment significance (* adjusted *p* < 0.05, ** adjusted *p* < 0.01, *** adjusted *p* < 0.001). Abbreviations: KEGG, Kyoto Encyclopedia of Genes and Genomes; SDMs, significant differential metabolites; DA, differential abundance; FDR, false discovery rate; CK, unchallenged control group; ACK, *A. hydrophila*-challenged control group.

**Figure 9:**
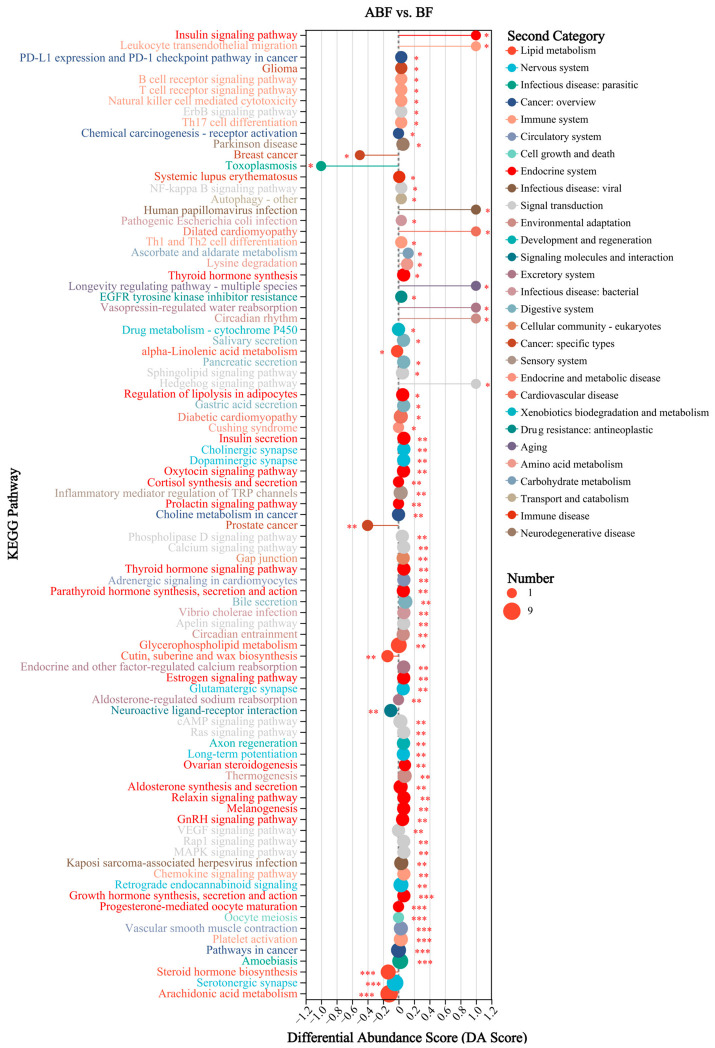
KEGG pathway enrichment analysis of hepatic SDMs in ABF versus BF after challenge. Dot size indicates the number of mapped metabolites, and colors indicate KEGG second-level categories. Positive and negative DA scores indicate overall metabolite increases and decreases in ABF relative to BF, respectively. Asterisks denote FDR-adjusted pathway enrichment significance (* adjusted *p* < 0.05, ** adjusted *p* < 0.01, *** adjusted *p* < 0.001). Abbreviations: KEGG, Kyoto Encyclopedia of Genes and Genomes; SDMs, significant differential metabolites; DA, differential abundance; FDR, false discovery rate; BF, unchallenged dietary *B. subtilis* Ya3.1-pretreated group; ABF, *A. hydrophila*-challenged dietary *B. subtilis* Ya3.1-pretreated group.

**Figure 10:**
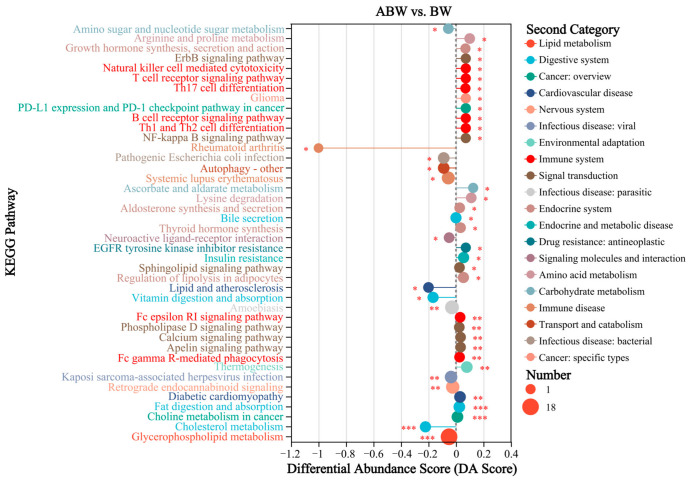
KEGG pathway enrichment analysis of hepatic SDMs in ABW versus BW after challenge. Dot size indicates the number of mapped metabolites, and colors indicate KEGG second-level categories. Positive and negative DA scores indicate overall metabolite increases and decreases in ABW relative to BW, respectively. Asterisks denote FDR-adjusted pathway enrichment significance (* adjusted *p* < 0.05, ** adjusted *p* < 0.01, *** adjusted *p* < 0.001). Abbreviations: KEGG, Kyoto Encyclopedia of Genes and Genomes; SDMs, significant differential metabolites; DA, differential abundance; FDR, false discovery rate; BW, unchallenged waterborne *B. subtilis* Ya3.1-pretreated group; ABW, *A. hydrophila*-challenged waterborne *B. subtilis* Ya3.1-pretreated group.

**Figure 11:**
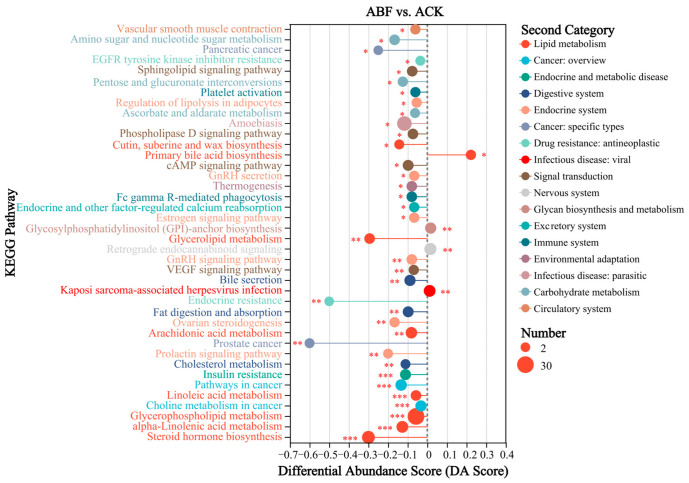
KEGG pathway enrichment analysis of hepatic SDMs in ABF versus ACK after challenge. Dot size indicates the number of mapped metabolites, and colors indicate KEGG second-level categories. Positive and negative DA scores indicate overall metabolite increases and decreases in ABF relative to ACK, respectively. Asterisks denote FDR-adjusted pathway enrichment significance (* adjusted *p* < 0.05, ** adjusted *p* < 0.01, *** adjusted *p* < 0.001). Abbreviations: KEGG, Kyoto Encyclopedia of Genes and Genomes; SDMs, significant differential metabolites; DA, differential abundance; FDR, false discovery rate; ACK, *A. hydrophila*-challenged control group; ABF, *A. hydrophila*-challenged dietary *B. subtilis* Ya3.1-pretreated group.

**Figure 12:**
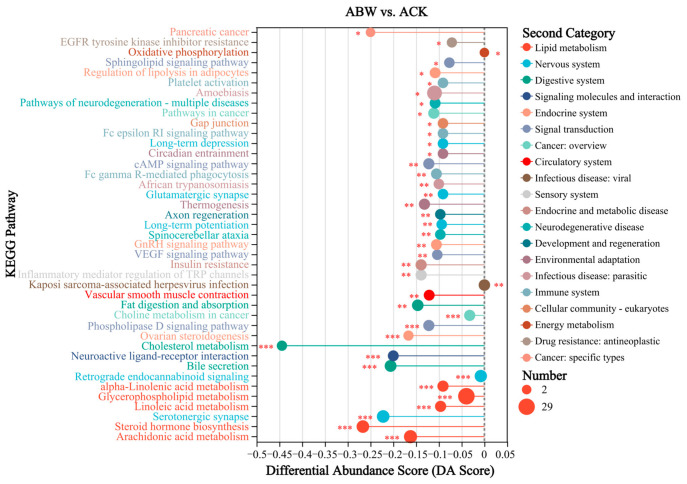
KEGG pathway enrichment analysis of hepatic SDMs in ABW versus ACK after challenge. Dot size indicates the number of mapped metabolites, and colors indicate KEGG second-level categories. Positive and negative DA scores indicate overall metabolite increases and decreases in ABW relative to ACK, respectively. Asterisks denote FDR-adjusted pathway enrichment significance (* adjusted *p* < 0.05, ** adjusted *p* < 0.01, *** adjusted *p* < 0.001). Abbreviations: KEGG, Kyoto Encyclopedia of Genes and Genomes; SDMs, significant differential metabolites; DA, differential abundance; FDR, false discovery rate; ACK, *A. hydrophila*-challenged control group; ABW, *A. hydrophila*-challenged waterborne *B. subtilis* Ya3.1-pretreated group.

KEGG pathway enrichment analysis of the significant differential metabolites was conducted to characterize functional metabolic changes (Figure 8, Figure 9, Figure 10, Figure 11 and Figure 12). Comparisons between pre- and post-challenge states identified significant enrichment of 74, 89, and 42 pathways in the control (CK vs. ACK; Figure 8), dietary pretreatment (BF vs. ABF; Figure 9), and waterborne pretreatment (BW vs. ABW; Figure 10) groups, respectively. Although numerous pathways were affected in all three comparisons, the absolute values of the differential abundance (DA) scores were generally lower in probiotic-pretreated fish than in the untreated control fish, suggesting a lower degree of pathway-level deviation after challenge.

When the challenged groups were compared directly, 40 and 41 pathways were significantly enriched in ABF vs. ACK and ABW vs. ACK, respectively (Figure 11 and Figure 12). Of these, 27 pathways were shared between the two probiotic delivery routes, indicating substantial overlap in the post-challenge hepatic metabolic responses associated with Ya3.1 pretreatment. In both comparisons, most shared pathways exhibited negative DA scores in the probiotic-pretreated groups relative to ACK, suggesting that the direction of pathway-level metabolic changes differed from that of the infected control state. Notably, among the recurrently enriched pathways, glycerophospholipid metabolism was consistently detected in both ABF vs. ACK and ABW vs. ACK, and it contained the largest number of differential metabolites in each comparison. Specifically, 30 glycerophospholipid metabolism-associated differential metabolites were identified in ABF vs. ACK, including 23 decreased and 7 increased metabolites. Similarly, 29 such metabolites were identified in ABW vs. ACK, including 20 decreased and 9 increased metabolites. To further characterize this pathway at the metabolite level, the glycerophospholipid metabolism-associated differential metabolites were ranked according to their absolute log2 FC, and the 10 top-ranked metabolites are summarized in Supplementary Table S5. These metabolites were mainly phosphatidylserines, phosphatidic acids, and phosphatidylethanolamine-related lipids, most of which showed lower abundance in the probiotic-pretreated challenged groups than in ACK. Taken together, the two Ya3.1 delivery routes showed overlapping probiotic-associated hepatic metabolic responses after *A. hydrophila* challenge, with glycerophospholipid metabolism emerging as a major candidate metabolic signature associated with Ya3.1-related post-challenge hepatic responses.

## 4. Discussion

### 4.1. Ya3.1-Associated Protection and Pathogen-Associated Intestinal Burden

Challenge trials provide a direct approach for evaluating fish disease resistance and overall health status [28,29]. In our previous 6-week feeding trial, pretreatment with the autochthonous probiotic *B. subtilis* Ya3.1 improved post-challenge survival of hybrid sturgeon following *A. hydrophila* infection [22]. Building on that previously reported protective phenotype, the present study further investigated the intestinal, humoral, and hepatic responses associated with Ya3.1-mediated protection. Absolute qPCR quantification of targeted bacterial marker genes showed that, before challenge, both dietary (BF) and waterborne (BW) Ya3.1 pretreatments resulted in significantly higher intestinal *B. subtilis*-associated gene copy numbers than those observed in CK. These results indicate that both delivery routes increased *B. subtilis*-related bacterial signals in the intestine, suggesting effective pre-challenge exposure of the host intestinal environment to Ya3.1. After challenge, the *A. hydrophila*-associated intestinal burden was significantly lower in ABF and ABW than in ACK, and both probiotic-pretreated groups showed significantly higher cumulative survival than ACK. These results indicate that Ya3.1 pretreatment was associated not only with a reduced pathogen-associated burden but also with a measurable host-level disease-resistance phenotype. Such a protective effect may be related to transient enrichment, adhesion, or residual persistence of the probiotic in the intestine, which could limit *A. hydrophila* expansion through ecological niche competition, nutrient competition, production of antimicrobial metabolites, modulation of the intestinal microbiota, reinforcement of mucosal barrier function, or activation of innate immune responses. These mechanisms are consistent with the recognized functions of probiotics in aquaculture, including microbial community modulation, pathogen suppression, immune enhancement, and improved disease resistance [10,30,31]. The observation that both dietary and waterborne pretreatments reduced pathogen-associated burden and improved survival suggests that both routes have practical application potential: dietary delivery may be more suitable for long-term, stable nutritional intervention, whereas waterborne delivery may provide a more practical preventive approach during periods of elevated pathogen-exposure risk in aquaculture systems.

### 4.2. Serum Innate Humoral Responses

The innate immune system constitutes a primary line of defense against pathogens in teleost fish, and humoral factors, such as complement components, lysozyme, and phosphatases, are commonly used as indicators of non-specific immunity and host protection [32]. In the present study, the serum innate immune assay results suggest that Ya3.1 pretreatment was associated with higher complement-related humoral indices before challenge in hybrid sturgeon exposed to *A. hydrophila* infection and with partial preservation of selected humoral immune indicators after challenge. Before challenge, Ya3.1 pretreatment significantly increased serum C3 in both delivery groups and C4 in the dietary group, suggesting a primary effect on complement-related humoral immunity. This finding is biologically relevant because the complement system is a central component of fish innate immunity, and C3 and C4 are closely involved in complement activation, pathogen recognition, opsonization, and downstream immune defense [33]. The elevation of C3 and C4 before challenge may therefore reflect a higher state of complement-associated immune readiness rather than a generalized activation of all measured immune factors. After challenge, AKP, ACP, C3, and C4 generally declined relative to their corresponding pre-challenge levels, which may reflect infection-associated immune suppression, inhibition of phosphatase activity, and/or complement consumption during bacterial clearance [34]. In contrast, the increase in LZM activity may represent a broad antibacterial response induced by pathogen stimulation [35]. Notably, ABF and ABW maintained significantly higher AKP activity than ACK at 10 dpi, and ABF also showed a significantly higher C3 concentration than ACK, indicating that Ya3.1 pretreatment partially preserved selected humoral indicators in surviving fish after infection. Similar immunomodulatory effects of *B. subtilis* have been reported in other fish species, where dietary supplementation improved serum lysozyme, alkaline phosphatase, antioxidant or complement-related immune indices, and enhanced resistance to *A. hydrophila* or other stressors [36,37,38]. Taken together, these findings support the view that Ya3.1-mediated protection was more likely to be associated with pre-challenge differences in complement-related humoral indices and post-challenge maintenance of selected serum defensive indicators, particularly AKP and C3.

### 4.3. Challenge-Associated Intestinal Microbiota Restructuring

The intestinal microbiota data provide complementary evidence that Ya3.1 pretreatment was associated with reduced infection-associated community displacement following pathogen challenge. Alpha-diversity analysis showed that the untreated challenged group (ACK) exhibited an increased Shannon index and a decreased Simpson index after challenge, suggesting that *A. hydrophila* infection may have disrupted the original dominance structure of the intestinal microbiota and shifted the community toward a more complex configuration. In contrast, ABF and ABW showed intermediate Shannon and Simpson values and were not significantly separated from their corresponding pre-challenge groups, indicating that Ya3.1 pretreatment may help buffer the impact of pathogen stimulation on intestinal microbial diversity. This interpretation was further supported by beta-diversity analysis: ACK samples were clearly separated from the other groups in both the PCA and Bray–Curtis PCoA plots, and their centroid displacement relative to the pre-challenge baseline was markedly greater than that of ABF and ABW. Meanwhile, PERMDISP detected no significant difference in within-group dispersion, suggesting that *A. hydrophila* infection mainly caused a directional shift in the overall community composition of untreated fish, whereas Ya3.1 pretreatment reduced this challenge-associated community displacement rather than simply altering between-sample variability. At the compositional level, ACK showed a decrease in the relative abundance of Firmicutes and increases in Proteobacteria, Actinobacteriota, and Bacteroidota after challenge, indicating a transition from a relatively Firmicutes-dominated profile to a more taxonomically heterogeneous community. At the genus level, ACK was characterized by increased relative abundances of several opportunistic or potentially pathogenic taxa, including *Hafnia-Obesumbacterium*, *Acinetobacter*, *Chitinophaga*, *Halomonas*, *Aeromonas*, and *Nesterenkonia*, whereas the dominant genera in the probiotic-pretreated groups remained comparatively stable before and after challenge. The enrichment of opportunistic taxa after bacterial challenge is consistent with previous reports that *A. hydrophila* infection can disturb microbial community structure and increase the relative abundance of potentially pathogenic or stress-associated bacteria in fish [39,40]. LEfSe analysis further showed that ACK, relative to CK, was enriched with several potentially opportunistic or dysbiosis-associated taxa, while no discriminatory genera were detected between BF and ABF or between BW and ABW. These results suggest that Ya3.1 pretreatment was associated with reduced challenge-associated restructuring of the intestinal microbiota. This is consistent with recent studies showing that *Bacillus*-based probiotics can improve intestinal microbial balance, intestinal health, and disease resistance in fish challenged with *A. hydrophila* [41,42]. Direct comparisons among the challenged groups also showed that ABF and ABW had lower relative abundances of several ACK-enriched opportunistic taxa, together with relative enrichment of *Bacillus*-associated lineages. This pattern may reflect the involvement of Ya3.1 in competitive exclusion, niche occupation, or indirect modulation of the host intestinal environment, mechanisms that have been proposed for probiotic-mediated protection in aquaculture species [41,43]. Overall, the effect of Ya3.1 on the intestinal microbiota of hybrid sturgeon is more appropriately interpreted as an association with reduced challenge-associated compositional restructuring. Under *A. hydrophila* infection pressure, Ya3.1 pretreatment appeared to limit the expansion of opportunistic taxa and maintain a community structure closer to the pre-challenge state.

### 4.4. Hepatic Metabolic Responses

The untargeted metabolomic results of the present study indicate that *B. subtilis* Ya3.1 pretreatment markedly modulated the hepatic metabolic status of hybrid sturgeon following *A. hydrophila* challenge. As the liver is a central organ for energy allocation, lipid metabolism, detoxification, and immune-stress regulation in fish, pathogen-induced hepatic metabolic alterations may reflect systemic physiological responses to bacterial stress [44,45]. In this study, clear separations were observed between ACK and CK, ABF and BF, and ABW and BW, indicating that *A. hydrophila* challenge was associated with substantial disturbance of hepatic metabolic homeostasis. Notably, both ABF and ABW were clearly separated from ACK after challenge, and the numbers of significant differential metabolites in the within-group pre- versus post-challenge comparisons were lower in the probiotic-pretreated groups than in the untreated control group. Although the number of differential metabolites should not be directly equated with disease severity or metabolic homeostasis, this pattern is consistent with a less extensive challenge-associated hepatic metabolic shift in Ya3.1-pretreated fish. These findings suggest that Ya3.1 may not simply prevent infection-induced metabolic changes, but may instead be associated with a modified host metabolic response to pathogen stimulation, resulting in a hepatic metabolic profile distinct from that of untreated infected fish. KEGG annotation further showed that the differential metabolites were mainly enriched in lipid-related classes, particularly phospholipid-associated metabolites, suggesting that lipid metabolism represents an important metabolic layer underlying the protective effect of Ya3.1. This interpretation is biologically plausible because phospholipids are not only fundamental structural components of cellular and organellar membranes, but also participate in membrane plasticity, cell signaling, immune-cell activation, oxidative stress responses, and inflammatory regulation [46,47]. Therefore, the recurrent enrichment of glycerophospholipid metabolism in both ABF vs. ACK and ABW vs. ACK, together with the lower abundance of multiple phosphatidylserine-, phosphatidic acid-, and phosphatidylethanolamine-related lipids in the probiotic-pretreated challenged groups, may reflect a candidate association with infection-induced membrane lipid remodeling and inflammation-associated lipid signaling. These interpretations remain hypothesis-generating because targeted lipidomics and functional membrane or inflammatory assays were not performed. This interpretation is also consistent with recent aquaculture studies showing that *B. subtilis* can improve liver health and regulate lipid metabolism in fish, and that probiotic interventions can affect host metabolite profiles, including glycerophospholipids and fatty acyl derivatives, during *A. hydrophila* challenge [43,48,49]. The two Ya3.1 delivery routes showed broadly similar pathway enrichment patterns, with 27 shared significantly enriched pathways between ABF vs. ACK and ABW vs. ACK, indicating that dietary and waterborne administration may induce partially conserved hepatic metabolic adaptations after bacterial challenge. Overall, these results support the conclusion that Ya3.1 pretreatment was associated with a distinct hepatic metabolic response of hybrid sturgeon under *A. hydrophila* infection, with glycerophospholipid-centered lipid metabolism emerging as a major candidate metabolic signature associated with Ya3.1-mediated protection.

### 4.5. Integrated Working Model

**Figure 13:**
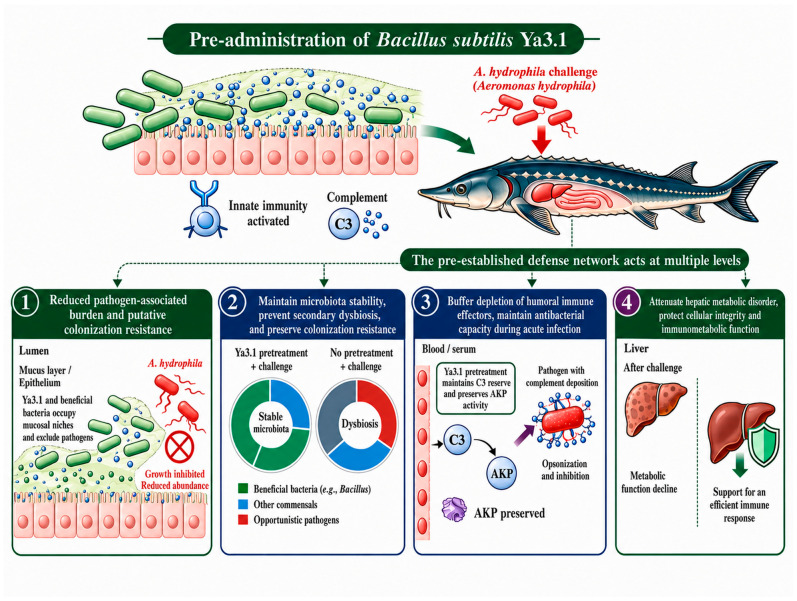
Hypothetical working model of *B. subtilis* Ya3.1-mediated protection against *A. hydrophila* in sturgeon.

Taken together, the multi-omics results support a hypothesis-generating working model, rather than a demonstrated causal mechanism, in which Ya3.1 pretreatment is associated with a more resilient host state before and during *A. hydrophila* challenge (Figure 13). This state is characterized by a lower pathogen-associated intestinal burden, reduced microbiota disruption, partial preservation of humoral immune indices, and a distinct hepatic metabolic response profile. Rather than demonstrating a single causal pathway, our data suggest that Ya3.1-associated protection may involve co-occurring changes in pathogen burden, microbial community composition, selected innate immune indices, and hepatic metabolic adaptation, which were accompanied by improved survival. Recent studies have emphasized that disease resistance in fish is shaped not by isolated processes but by the dynamic interplay among mucosal barriers, microbial communities, immune networks, and metabolic resources [4,50,51,52,53]. Our findings are consistent with this systems-level framework and further underscore the value of integrating microbiological, immunological, and metabolomic datasets to elucidate the modes of action of probiotics in aquatic animals [24,54].

### 4.6. Limitations

Several limitations should be considered when interpreting the present findings. First, although the intraperitoneal injection model provided standardized infection pressure and reproducible survival comparisons, it does not fully reflect natural oral or immersion exposure to *A. hydrophila* and thus may not capture farm-relevant pathogen colonization, epithelial invasion, or mucosal immune responses. Second, the lack of a PBS-injected sham control limited the ability to distinguish pathogen-induced effects from injection- or handling-related stress, while the absence of a continuous-administration comparison group prevented clear separation of residual Ya3.1 pretreatment effects from possible withdrawal-associated changes in microbiota or immunity. Third, targeted qPCR quantified *B. subtilis*- and *A. hydrophila*-associated marker gene copies rather than viable cells or strain-specific signals; notably, the *B. subtilis* assay could not distinguish the administered Ya3.1 strain from endogenous *Bacillus* populations. Fourth, terminal sampling was performed only at 10 days post-infection and only in surviving fish; therefore, the observed serum immune, microbiota, and metabolomic profiles should be regarded as survivor-associated endpoint states rather than dynamic host-response trajectories or responses in fish that succumbed to infection before terminal sampling. Fifth, serum AKP, ACP, C3, and C4 are relatively non-specific immune indicators and should not be interpreted as direct evidence of broadly enhanced immune competence. In addition, intestinal barrier function was not directly evaluated by histology, tight-junction gene expression, mucus-related indices, or permeability assays, leaving mucosal barrier-related mechanisms inferential. Finally, the microbiota and metabolomic data provide associative rather than causal evidence, and the relatively small sample sizes used for these analyses further limit mechanistic interpretation. Future studies using natural-route infection models, sham controls, continuous-administration comparisons, longitudinal sampling, strain-specific tracking, viable bacterial enumeration, mucosal barrier assays, larger multi-omics cohorts, and functional validation approaches are needed to clarify the mechanisms underlying Ya3.1-mediated protection.

## 5. Conclusions

In conclusion, this integrative follow-up study provides evidence that the previously reported protective effect of the autochthonous probiotic *B. subtilis* Ya3.1 against *A. hydrophila* infection in hybrid sturgeon is associated with coordinated changes in the gut microbiota, selected humoral immune indices, and hepatic metabolism. After prior exposure via either feed or the rearing water, Ya3.1-pretreated fish showed a lower intestinal *A. hydrophila*-associated burden, reduced challenge-associated displacement of the intestinal microbiota, partial preservation of selected humoral immune indices, and a distinct hepatic metabolic profile after challenge. Glycerophospholipid metabolism emerged as a recurrent enriched pathway across both delivery routes, suggesting a candidate metabolic feature that correlates with regulation of host immunometabolic homeostasis under Ya3.1 pretreatment. These findings extend our previous efficacy observations by providing multilayer, hypothesis-generating evidence for host and microbial responses associated with Ya3.1 pretreatment. Future studies using strain-specific tracking, longitudinal immune sampling, targeted lipidomics, and functional validation are warranted to clarify the causal mechanisms involved.

## Figures and Tables

**Table 1:** Alpha diversity indices of the intestinal microbiota before and after the challenge.

Parameters	Pre-Challenge			Post-Challenge		
	CK	BF	BW	ACK	ABF	ABW
Sobs	125.33 ± 20.33	138.67 ± 10.23	146.83 ± 9.84	195.33 ± 11.78	187.83 ± 18.42	192.33 ± 24.71
ACE	156.85 ± 20.05	175.19 ± 13.39	167.79 ± 12.65	207.43 ± 10.36	205.67 ± 16.80	215.92 ± 26.94
Chao1	150.21 ± 20.01	173.75 ± 15.23	166.67 ± 14.25	206.34 ± 10.34	203.65 ± 17.82	213.23 ± 27.92
Simpson	0.62 ± 0.05 ^b^	0.69 ± 0.04 ^b^	0.45 ± 0.09 ^ab^	0.26 ± 0.02 ^a^	0.57 ± 0.08 ^ab^	0.50 ± 0.08 ^ab^
Shannon	0.80 ± 0.07 ^a^	0.77 ± 0.09 ^a^	1.39 ± 0.23 ^ab^	2.01 ± 0.11 ^b^	1.14 ± 0.22 ^ab^	1.36 ± 0.23 ^ab^

Values are presented as the mean ± SE (*n* = 6 fish per group). Different lowercase letters within the same row indicate significant differences among experimental groups (adjusted *p* < 0.05). Abbreviations: Sobs, observed operational taxonomic units; ACE, abundance-based coverage estimator; CK, unchallenged control group; BF, unchallenged dietary *B. subtilis* Ya3.1-pretreated group; BW, unchallenged waterborne *B. subtilis* Ya3.1-pretreated group; ACK, *A. hydrophila*-challenged control group; ABF, *A. hydrophila*-challenged dietary *B. subtilis* Ya3.1-pretreated group; ABW, *A. hydrophila*-challenged waterborne *B. subtilis* Ya3.1-pretreated group.

## Data Availability

The 16S rRNA gene sequencing data are available in the NCBI Sequence Read Archive under BioProjects PRJNA1370269 and PRJNA1455859. Additional data supporting the findings of this study are available from the corresponding author on reasonable request.

## Supplementary Materials

The following supporting information can be downloaded at: https://www.mdpi.com/article/10.3390/ani16121879/s1, Table S1: Sample-specific 16S rRNA gene sequencing quality metrics. Table S2: Pairwise PERMANOVA comparisons of intestinal microbial community structure. Table S3: Bray–Curtis centroid-displacement and PERMDISP analyses of intestinal microbiota composition. Table S4: Functional classification of KEGG-annotated significant differential metabolites. Table S5: Top 10 glycerophospholipid metabolism-associated differential metabolites and fold changes in ABF versus ACK and ABW versus ACK.
