# Supplementary material for: Protective Effects and Potential Mechanisms of Bacillus subtilis Ya3.1 Against Aeromonas hydrophila Infection in Hybrid Sturgeon (Acipenser schrenckii ♂ × Huso dauricus ♀)
Source: Animals (Basel). 2026 Jun 17;16(12):1879. doi: 10.3390/ani16121879 (PMC13295440; doi:10.3390/ani16121879)
Supplement: Supplementary file 1 [file animals-16-01879-s001.zip › animals-4358455-supplementary.pdf]

Supplementary Materials

Protective Effects and Potential Mechanisms of *Bacillus subtilis* Ya3.1 against *Aeromonas hydrophila* Infection in Hybrid Sturgeon (*Acipenser schrenckii* ♂ × *Huso dauricus* ♀)

Wei Huang [1,2,†], Yang Liu [1,2,†], Lanyin Liu [1], Xin Lv [1], Yongkang Song [1] and Tuyan Luo [1,2,*]

1 Institute of Agricultural Quality Standards and Testing Technology Research, Fujian Academy of Agricultural Sciences, No.247, Wusi Road, Gulou District, Fuzhou, Fujian 350003, China
2 Fujian Key Laboratory of Agro-products Quality and Safety, Fujian Academy of Agricultural Sciences, No.247, Wusi Road, Gulou District, Fuzhou, Fujian 350003, China
† These authors contributed equally to this work.
* Correspondence: luotuyan@faas.cn (T.L.)

Table S1. Sample-specific 16S rRNA gene sequencing quality metrics.

Sample ID	Raw reads (n)	Clean reads (n)	Read retention (%)	Good's coverage (%)
CK1	112,137	108,238	96.52	99.98
CK2	120,434	116,545	96.77	99.98
CK3	307,758	267,880	87.04	99.98
CK4	249,452	216,568	86.82	99.99
CK5	61,932	56,140	90.65	99.96
CK6	228,682	181,719	79.46	99.96
BF1	112,601	105,740	93.91	99.97
BF2	108,944	104,372	95.80	99.97
BF3	77,187	77,029	99.80	99.98
BF4	104,895	102,449	97.67	99.96
BF5	114,317	110,625	96.77	99.98
BF6	202,412	193,931	95.81	99.98
BW1	45,591	39,392	86.40	99.96
BW2	151,658	147,402	97.19	99.99
BW3	79,041	73,208	92.62	99.96
BW4	176,329	170,268	96.56	99.98
BW5	118,203	113,434	95.97	99.99
BW6	100,643	99,166	98.53	99.98
ACK1	129,665	102,485	79.04	99.99
ACK2	129,620	86,873	67.02	99.97
ACK3	89,919	63,308	70.41	99.81
ACK4	83,490	54,686	65.50	99.95
ACK5	83,490	54,665	65.47	99.93
ACK6	176,643	156,307	88.49	99.98
ABF1	146,114	124,170	84.98	99.99
ABF2	150,144	109,689	73.06	99.98
ABF3	153,555	115,790	75.41	99.97
ABF4	152,055	98,808	64.98	99.98
ABF5	298,176	257,108	86.23	99.98
ABF6	129,315	118,538	91.67	99.99
ABW1	211,592	176,431	83.38	99.98
ABW2	189,538	128,534	67.81	99.97
ABW3	158,320	130,219	82.25	99.99
ABW4	112,874	101,037	89.51	99.98
ABW5	59,243	58,031	97.95	99.97
ABW6	112,025	106,149	94.75	99.99

Read retention (%) was calculated as clean reads/raw reads × 100. Good's coverage was used to estimate sequencing completeness. Each sample ID represents one intestinal sample; two fish were sampled from each replicate tank (n = 6 fish per group). Abbreviations: CK, unchallenged control group; BF, unchallenged dietary *B. subtilis* Ya3.1-pretreated group; BW, unchallenged waterborne *B. subtilis* Ya3.1-pretreated group; ACK, *A. hydrophila*-challenged control group; ABF, *A. hydrophila*-challenged dietary *B. subtilis* Ya3.1-pretreated group; ABW, *A. hydrophila*-challenged waterborne *B. subtilis* Ya3.1-pretreated group.

Table S2. Pairwise and overall PERMANOVA results based on Bray–Curtis dissimilarity matrices comparing intestinal microbial community composition among experimental groups.

Comparison	n	Groups	F	R^2	*P* value	Adjusted *P* value
Overall						
All groups	36	6	19.966	0.769	0.001	
Pre-challenge comparisons						
BW vs BF	12	2	5.969	0.374	0.038	0.077
CK vs BF	12	2	3.738	0.272	0.052	0.087
CK vs BW	12	2	2.901	0.225	0.075	0.102
Post-challenge comparisons						
ABF vs ABW	12	2	1.187	0.106	0.307	0.329
**ABF vs ACK**	**12**	**2**	**55.261**	**0.847**	**0.003**	**0.011**
**ABW vs ACK**	**12**	**2**	**38.903**	**0.796**	**0.002**	**0.010**
Challenge effect (post- vs pre-challenge controls)						
**ACK vs CK**	**12**	**2**	**95.779**	**0.905**	**0.002**	**0.010**
Probiotic preservation (post- vs pre-challenge probiotic groups)						
**ABW vs BW**	**12**	**2**	**0.326**	**0.032**	**0.845**	**0.845**
**ABF vs BF**	**12**	**2**	**3.308**	**0.249**	**0.060**	**0.090**
Cross-group comparisons						
**ACK vs BF**	**12**	**2**	**147.053**	**0.936**	**0.004**	**0.012**
**ACK vs BW**	**12**	**2**	**44.852**	**0.818**	**0.002**	**0.010**
ABF vs CK	12	2	2.971	0.229	0.041	0.077
ABF vs BW	12	2	1.955	0.164	0.163	0.204
ABW vs BF	12	2	4.362	0.304	0.013	0.032
ABW vs CK	12	2	1.537	0.133	0.210	0.242

Pseudo-F represents the model F statistic, and R^2 indicates the proportion of variance explained by the grouping factor. Abbreviations: PERMANOVA, permutational multivariate analysis of variance; group abbreviations are as defined in Table S1.

Table S3. Bray–Curtis centroid-displacement and PERMDISP analyses of intestinal microbiota composition.

A. Bray–Curtis centroid displacement from each post-challenge sample to the centroid of its corresponding pre-challenge baseline group.

Post-challenge group	Baseline centroid	n	Mean ± SE	Median	Reduction relative to ACK
ACK	CK	6	0.8644 ± 0.0251	0.8439	0%
ABF	BF	6	0.2202 ± 0.0507	0.1956	74.52%
ABW	BW	6	0.2154 ± 0.0509	0.1737	75.08%

B. Statistical comparison of centroid displacement among challenged groups.

Test / comparison	Statistic	*P* value	Adjusted *P* value	Interpretation
Kruskal–Wallis	H = 11.4152	0.00332	–	Significant among challenged groups
ACK vs. ABF	Mann–Whitney U	0.00216	0.00649	ABF < ACK
ACK vs. ABW	Mann–Whitney U	0.00216	0.00649	ABW < ACK
ABF vs. ABW	Mann–Whitney U	0.81818	0.81818	Not significant

C. PERMDISP/betadisper multivariate dispersion analysis based on the Bray–Curtis distance matrix.

Analysis	Group / Comparison	Result
PERMDISP dispersion	CK	0.1622 ± 0.0096
PERMDISP dispersion	BF	0.1011 ± 0.0048
PERMDISP dispersion	BW	0.2100 ± 0.0368
PERMDISP dispersion	ACK	0.1753 ± 0.0090
PERMDISP dispersion	ABF	0.1819 ± 0.0288
PERMDISP dispersion	ABW	0.2135 ± 0.0475
PERMDISP overall test	Six groups	F = 2.1667, permutation *P* = 0.5598

Centroid displacement was calculated as the Bray–Curtis distance from each post-challenge sample to the centroid of its corresponding pre-challenge baseline group: ACK to the CK centroid, ABF to the BF centroid, and ABW to the BW centroid. Centroid-displacement and PERMDISP dispersion values are presented as mean ± SE. Abbreviations: SE, standard error; PERMDISP, permutational analysis of multivariate dispersions; group abbreviations are as defined in Table S1.

Table S4. Functional classification of KEGG-annotated significant differential metabolites (SDMs) across pairwise comparisons.

Comparison	Total SDMs	KEGG-annotated SDMs	KEGG annotation coverage (%)	Not KEGG-classified SDMs	Lipids n (%)	Amino acids n (%)	Carbohydrates n (%)	Vitamins/cofactors n (%)	Steroids/hormones/transmitters n (%)	Nucleic acids n (%)	Organic acids n (%)	Antibiotics/secondary metabolites n (%)
ACK vs. CK	470	47	10.0%	423	40 (85.1%)	0 (0.0%)	0 (0.0%)	1 (2.1%)	4 (8.5%)	1 (2.1%)	0 (0.0%)	1 (2.1%)
ABF vs. BF	317	27	8.5%	290	15 (55.6%)	0 (0.0%)	2 (7.4%)	0 (0.0%)	8 (29.6%)	1 (3.7%)	0 (0.0%)	1 (3.7%)
ABW vs. BW	280	30	10.7%	250	20 (66.7%)	3 (10.0%)	2 (6.7%)	3 (10.0%)	0 (0.0%)	0 (0.0%)	0 (0.0%)	2 (6.7%)
ABF vs. ACK	623	67	10.8%	556	42 (62.7%)	0 (0.0%)	2 (3.0%)	1 (1.5%)	16 (23.9%)	0 (0.0%)	0 (0.0%)	6 (9.0%)
ABW vs. ACK	453	58	12.8%	395	42 (72.4%)	1 (1.7%)	1 (1.7%)	0 (0.0%)	13 (22.4%)	0 (0.0%)	0 (0.0%)	1 (1.7%)

Total SDMs were taken from the corresponding SDM screening results shown in the volcano plots. Functional-class percentages were calculated using KEGG-annotated SDMs as the denominator. Unannotated or non-KEGG-classified SDMs were not assigned to functional classes to avoid overinterpretation. The Lipids column follows the KEGG first-category annotation and is reported separately from steroids/hormones/transmitters. Antibiotics/secondary metabolites refers to the KEGG chemical classification category, not antibiotic treatment. Classification rule: peptides/amino acids were assigned to amino acids; steroids and hormones and transmitters were grouped as steroids/hormones/transmitters; all remaining categories followed their KEGG first-category names unless otherwise stated. Abbreviations: SDMs, significant differential metabolites; KEGG, Kyoto Encyclopedia of Genes and Genomes; group abbreviations are as defined in Table S1.

Table S5. Top glycerophospholipid metabolism-associated differential metabolites in ABF versus ACK and ABW versus ACK.

A. ABF vs. ACK: top 10 metabolites ranked by absolute log_2FC.

Rank	Metabolite	HMDB subclass	FC	log_2FC	VIP	*P* value	Adjusted *P* value	Regulation
1	PS(22:6(4Z,7Z,10Z,13Z,16Z,19Z)/20:2(11Z,14Z))	Glycerophosphoserines	0.2718	-1.8794	1.9582	0.0137	0.0176	Down
2	PS(18:1(11Z)/14:0)	Glycerophosphoserines	0.2785	-1.8443	3.2186	0.0031	0.0048	Down
3	PS(22:5(7Z,10Z,13Z,16Z,19Z)/22:5(7Z,10Z,13Z,16Z,19Z))	Glycerophosphoserines	0.4452	-1.1675	2.5397	8.063E-06	0.0032	Down
4	PA(8:0/10:0)	Glycerophosphates	0.5053	-0.9848	2.4291	2.501E-05	0.0065	Down
5	LysoPC(17:0/0:0)	Glycerophosphocholines	0.607	-0.7202	1.9912	0.0183	0.0476	Down
6	PS(22:6(4Z,7Z,10Z,13Z,16Z,19Z)/22:6(4Z,7Z,10Z,13Z,16Z,19Z))	Glycerophosphoserines	0.6207	-0.688	1.8364	0.0189	0.0214	Down
7	PS(18:3(9Z,12Z,15Z)/18:1(11Z))	Glycerophosphoserines	0.63	-0.6666	2.1768	0.0272	0.0436	Down
8	PE-NMe2(20:5(5Z,8Z,11Z,14Z,17Z)/14:1(9Z))	Glycerophosphoethanolamines	0.6313	-0.6636	2.3134	0.0032	0.0479	Down
9	PS(22:5(7Z,10Z,13Z,16Z,19Z)/16:0)	Glycerophosphoserines	0.6645	-0.5897	2.2292	0.0103	0.0332	Down
10	PA(8:0/i-12:0)	Glycerophosphates	0.6844	-0.5471	1.7489	0.0013	0.0329	Down

B. ABW vs. ACK: top 10 metabolites ranked by absolute log_2FC.

Rank	Metabolite	HMDB subclass	FC	log_2FC	VIP	*P* value	Adjusted *P* value	Regulation
1	PS(18:1(11Z)/14:0)	Glycerophosphoserines	0.2647	-1.9176	3.9765	0.0022	0.0145	Down
2	CDP-DG(i-16:0/i-18:0)	CDP-glycerols	0.4717	-1.0841	2.5141	0.0089	0.0171	Down
3	PA(8:0/10:0)	Glycerophosphates	0.5249	-0.9299	3.0202	2.625E-04	0.0495	Down
4	PS(22:5(7Z,10Z,13Z,16Z,19Z)/22:5(7Z,10Z,13Z,16Z,19Z))	Glycerophosphoserines	0.5669	-0.8188	2.5658	0.0022	0.0146	Down
5	PS(22:6(4Z,7Z,10Z,13Z,16Z,19Z)/22:6(4Z,7Z,10Z,13Z,16Z,19Z))	Glycerophosphoserines	0.5803	-0.7851	2.3461	0.0118	0.0218	Down
6	PG(22:6(4Z,7Z,10Z,13Z,16Z,19Z)/20:4(8Z,11Z,14Z,17Z))	Glycerophosphoglycerols	1.5164	0.6007	2.3854	0.0121	0.0182	Up
7	PS(22:4(7Z,10Z,13Z,16Z)/22:5(7Z,10Z,13Z,16Z,19Z))	Glycerophosphoserines	0.6746	-0.5679	1.7519	0.039	0.0463	Down
8	PS(22:6(4Z,7Z,10Z,13Z,16Z,19Z)/22:2(13Z,16Z))	Glycerophosphoserines	0.7238	-0.4663	1.6669	0.03	0.0435	Down
9	PE-NMe(16:1(9Z)/20:5(5Z,8Z,11Z,14Z,17Z))	Glycerophosphoethanolamines	0.7241	-0.4657	1.8236	0.0307	0.0381	Down
10	PS(22:5(7Z,10Z,13Z,16Z,19Z)/16:0)	Glycerophosphoserines	0.7334	-0.4473	2.2587	0.0213	0.0428	Down

Metabolites were ranked by absolute log_2FC. FC represents ABF/ACK or ABW/ACK. FC < 1 indicates lower abundance in the probiotic-pretreated challenged group than in ACK; FC > 1 indicates higher abundance. Abbreviations: HMDB, Human Metabolome Database; FC, fold change; VIP, variable importance in projection; group abbreviations are as defined in Table S1.
